# *trans*-Chlorido{3-chloro-2-[(1-naphth­yl)imino­meth­yl]phenyl-κ^2^ *C* ^1^,*N*}bis­(trimethyl­phosphane)nickel(II)

**DOI:** 10.1107/S1600536810046234

**Published:** 2010-11-17

**Authors:** Ruixia Cao, Hongjian Sun

**Affiliations:** a Department of Chemistry, Qilu Normal University, Jinan 250013, People’s Republic of China; b School of Chemistry and Chemical Engineering, Shandong University, Jinan 250100, People’s Republic of China

## Abstract

The title compound, [Ni(C_17_H_11_ClN)Cl(C_3_H_9_P)_2_], was obtained as a product of the reaction of [Ni(PMe_3_)_4_] with a molar equivalent of 2,6-dichloro-*N*-naphthyl­benzaldehyde­amine in diethyl ether. The τ parameter is 0.3, indicating that the coordination geometry is square-pyramidal. The Ni^II^ atom lies in the center of a square pyramidal in which one C, one Cl and two P atoms form the basal plane, with the imine N atom in an apical position. Two P-atom donors are located in *trans* positions.

## Related literature

For related structures of nickel compounds, see: Cao *et al.* (2008 ▶). For the τ parameter, see: Addison *et al.* (1984 ▶). 
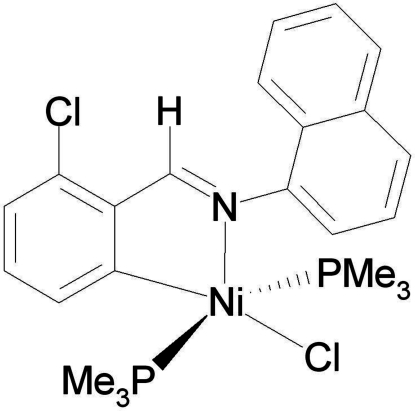


## Experimental

### 

#### Crystal data

[Ni(C_17_H_11_ClN)Cl(C_3_H_9_P)_2_]
*M* *_r_* = 511.02
Orthorhombic, 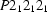

*a* = 9.0529 (19) Å
*b* = 15.855 (3) Å
*c* = 17.869 (4) Å
*V* = 2564.7 (9) Å^3^
*Z* = 4
Mo *K*α radiation
μ = 1.10 mm^−1^
*T* = 273 K
0.12 × 0.10 × 0.08 mm

#### Data collection

Bruker APEXII CCD diffractometer
Absorption correction: multi-scan (*SADABS*; Bruker, 2001 ▶) *T*_min_ = 0.879, *T*_max_ = 0.917
9377 measured reflections
3708 independent reflections
3376 reflections with *I* > 2σ(*I*)
*R*_int_ = 0.045
θ_max_ = 23.5°

#### Refinement

*R*[*F* ^2^ > 2σ(*F* ^2^)] = 0.036
*wR*(*F* ^2^) = 0.124
*S* = 1.00
3708 reflections
268 parameters
H-atom parameters constrained
Δρ_max_ = 0.30 e Å^−3^
Δρ_min_ = −0.24 e Å^−3^
Absolute structure: Flack (1983 ▶), 1893 Friedel pairs
Flack parameter: −0.03 (2)

### 

Data collection: *APEX2* (Bruker, 2004 ▶); cell refinement: *SAINT-Plus* (Bruker, 2001 ▶); data reduction: *SAINT-Plus*; program(s) used to solve structure: *SHELXS97* (Sheldrick, 2008 ▶); program(s) used to refine structure: *SHELXL97* (Sheldrick, 2008 ▶); molecular graphics: *SHELXTL* (Sheldrick, 2008 ▶); software used to prepare material for publication: *SHELXTL*.

## Supplementary Material

Crystal structure: contains datablocks global, I. DOI: 10.1107/S1600536810046234/bv2163sup1.cif

Structure factors: contains datablocks I. DOI: 10.1107/S1600536810046234/bv2163Isup2.hkl

Additional supplementary materials:  crystallographic information; 3D view; checkCIF report

## Figures and Tables

**Fig. 1.**
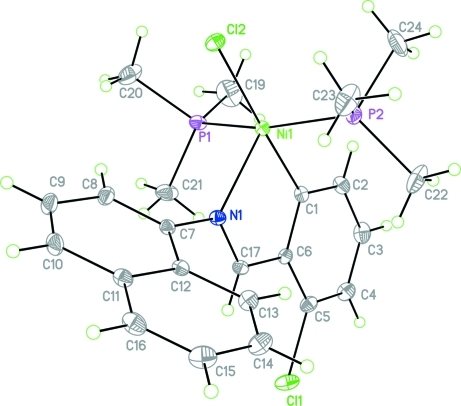
A view of the structure of (I), showing the atmoic numbering scheme and 30% probability displacement ellipsoids.

## supplementary crystallographic information

### Comment

In the title molecule (Fig.1) the nickel atom lies in the center of a square pyramidal geometry (τ parameter is 0.3, Addison *et al.* 1984) in which C, Cl and two P atoms form the basal plane withthe imine N in the apical position. Two P-donor atoms are located in *trans*-positions. A five membered metallacycle is formed through the coordination of the N atom of the imine group and the *ortho*-chelated C atom. The sum of internal bond angles (540%A) of this chelate ring indicates ideal planarity. The bite angle of the chelating ligand [C1—Ni1—N1 = 80.63 (15) %A] is close to that recently reported (Cao *et al.*, 2008). Similar crystal structures been reported in the literature *e.g.* N–(*o*-chlorine-phenyl)-2,6-dichlorobenzaldehydeamine-*trans*-b is(trimethylphosphine)nickel(II) (Cao *et al.*, 2008). The benzene plane forms an angle of 72.3 (1)%A with five membered metallacycle, which is smaller than the title compound (76.2 (1)%A). The bond lengths and angles of this compound are similar to those in the title compound.

### Experimental

A sample of Ni(PMe_3_)_4_ (1.0 g, 2.75 mmol) in 30 ml of diethyl ether was combined with a solution of *N*-naphthyl-2,6-dichlorobenzaldehydeamine (0.83 g, 2.75 mmol) in diethyl ether (20 ml) at -80%A. The reaction mixture was warmed to ambient temperature and stirred for 18 h to form a brown-yellow solution. The volatiles were removed *in vacuo*, and the resulting solid was extracted with pentane (60 ml). Crystallization at 4%A afforded brown-yellow crystals suitable for X-ray diffraction analysis (yield 0.59 g, 42%), Mp: 146%A.

### Refinement

All H atoms on C were placed in calculated positions with a C—H bond distance of 0.93 or 0.96 Å and *U*_iso_(H) = 1.2*U*_eq_ of the carrier atom.

### Crystal data

**Table:**

[Ni(C_17_H_11_ClN)Cl(C_3_H_9_P)_2_]	*F*(000) = 1064
*M**_r_* = 511.02	*D*_x_ = 1.323 Mg m^−^^3^
Orthorhombic, *P*2_1_2_1_2_1_	Mo *K*α radiation, λ = 0.71073 Å
Hall symbol: P 2ac 2ab	Cell parameters from 5708 reflections
*a* = 9.0529 (19) Å	θ = 3.5–27.2°
*b* = 15.855 (3) Å	µ = 1.10 mm^−^^1^
*c* = 17.869 (4) Å	*T* = 273 K
*V* = 2564.7 (9) Å^3^	Block, brown
*Z* = 4	0.12 × 0.10 × 0.08 mm

### Data collection

**Table:**

Bruker APEXII CCD diffractometer	3708 independent reflections
Radiation source: fine-focus sealed tube	3376 reflections with *I* > 2σ(*I*)
graphite	*R*_int_ = 0.045
phi and ω scans	θ_max_ = 23.5°, θ_min_ = 3.5°
Absorption correction: multi-scan (*SADABS*; Bruker, 2001)	*h* = −7→9
*T*_min_ = 0.879, *T*_max_ = 0.917	*k* = −16→17
9377 measured reflections	*l* = −20→14

### Refinement

**Table:**

Refinement on *F*^2^	Secondary atom site location: difference Fourier map
Least-squares matrix: full	Hydrogen site location: inferred from neighbouring sites
*R*[*F*^2^ > 2σ(*F*^2^)] = 0.036	H-atom parameters constrained
*wR*(*F*^2^) = 0.124	*w* = 1/[σ^2^(*F*_o_^2^) + (0.1*P*)^2^ + 0.1*P*] where *P* = (*F*_o_^2^ + 2*F*_c_^2^)/3
*S* = 1.00	(Δ/σ)_max_ = 0.077
3708 reflections	Δρ_max_ = 0.30 e Å^−^^3^
268 parameters	Δρ_min_ = −0.23 e Å^−^^3^
0 restraints	Absolute structure: Flack (1983), 1530 Friedel pairs
Primary atom site location: structure-invariant direct methods	Flack parameter: −0.03 (2)

### Special details

**Table:**

Geometry. All e.s.d.'s (except the e.s.d. in the dihedral angle between two l.s. planes) are estimated using the full covariance matrix. The cell e.s.d.'s are taken into account individually in the estimation of e.s.d.'s in distances, angles and torsion angles; correlations between e.s.d.'s in cell parameters are only used when they are defined by crystal symmetry. An approximate (isotropic) treatment of cell e.s.d.'s is used for estimating e.s.d.'s involving l.s. planes.
Refinement. Refinement of *F*^2^ against ALL reflections. The weighted *R*-factor *wR* and goodness of fit *S* are based on *F*^2^, conventional *R*-factors *R* are based on *F*, with *F* set to zero for negative *F*^2^. The threshold expression of *F*^2^ > σ(*F*^2^) is used only for calculating *R*-factors(gt) *etc*. and is not relevant to the choice of reflections for refinement. *R*-factors based on *F*^2^ are statistically about twice as large as those based on *F*, and *R*- factors based on ALL data will be even larger.

### Fractional atomic coordinates and isotropic or equivalent isotropic displacement parameters (Å^2^)

**Table:**

	*x*	*y*	*z*	*U*_iso_*/*U*_eq_	
Ni1	0.24052 (6)	0.05187 (3)	0.99453 (3)	0.03814 (19)	
P1	0.35371 (15)	0.15563 (8)	1.05246 (8)	0.0494 (3)	
Cl2	0.17839 (16)	0.13255 (7)	0.89625 (6)	0.0534 (3)	
P2	0.06670 (16)	−0.03809 (9)	0.96442 (8)	0.0553 (4)	
Cl1	0.6167 (2)	−0.17658 (10)	1.12882 (9)	0.0768 (5)	
C1	0.2869 (5)	−0.0146 (3)	1.0792 (2)	0.0414 (11)	
C11	0.6215 (6)	−0.0827 (3)	0.7665 (3)	0.0499 (12)	
C7	0.5207 (5)	−0.0170 (3)	0.8785 (2)	0.0418 (11)	
C6	0.4108 (5)	−0.0671 (3)	1.0723 (2)	0.0407 (10)	
C5	0.4571 (6)	−0.1144 (3)	1.1346 (3)	0.0531 (13)	
C12	0.5413 (5)	−0.0902 (3)	0.8345 (2)	0.0415 (11)	
C13	0.4820 (6)	−0.1712 (3)	0.8525 (3)	0.0549 (13)	
H13	0.4258	−0.1778	0.8956	0.066*	
C16	0.6432 (6)	−0.1554 (4)	0.7219 (3)	0.0677 (16)	
H16	0.6961	−0.1509	0.6775	0.081*	
C3	0.2568 (7)	−0.0621 (4)	1.2072 (3)	0.0676 (15)	
H3	0.2048	−0.0611	1.2520	0.081*	
C8	0.5727 (6)	0.0592 (3)	0.8552 (3)	0.0540 (12)	
H8	0.5541	0.1072	0.8837	0.065*	
C10	0.6795 (8)	−0.0025 (4)	0.7460 (3)	0.0629 (16)	
H10	0.7354	0.0027	0.7025	0.076*	
C9	0.6544 (6)	0.0660 (4)	0.7886 (3)	0.0636 (15)	
H9	0.6914	0.1181	0.7739	0.076*	
C4	0.3816 (7)	−0.1120 (4)	1.2009 (3)	0.0625 (15)	
H4	0.4139	−0.1438	1.2414	0.075*	
C2	0.2089 (6)	−0.0139 (3)	1.1482 (3)	0.0549 (13)	
H2	0.1248	0.0193	1.1533	0.066*	
C15	0.5889 (8)	−0.2305 (4)	0.7425 (3)	0.0728 (18)	
H15	0.6066	−0.2776	0.7129	0.087*	
C14	0.5063 (7)	−0.2384 (3)	0.8077 (3)	0.0656 (17)	
H14	0.4673	−0.2907	0.8207	0.079*	
C17	0.4894 (5)	−0.0668 (3)	1.0012 (2)	0.0417 (10)	
H17	0.5725	−0.1003	0.9943	0.050*	
N1	0.4418 (4)	−0.0194 (2)	0.9485 (2)	0.0408 (9)	
C20	0.3896 (9)	0.2490 (4)	0.9963 (5)	0.101 (3)	
H20A	0.2987	0.2786	0.9877	0.151*	
H20B	0.4317	0.2325	0.9492	0.151*	
H20C	0.4574	0.2852	1.0223	0.151*	
C22	0.0628 (10)	−0.1413 (4)	1.0068 (4)	0.100 (3)	
H22A	0.0305	−0.1366	1.0578	0.150*	
H22B	0.1600	−0.1655	1.0055	0.150*	
H22C	−0.0043	−0.1769	0.9797	0.150*	
C19	0.2610 (11)	0.2004 (5)	1.1325 (5)	0.125 (3)	
H19A	0.2614	0.1603	1.1728	0.188*	
H19B	0.1608	0.2138	1.1194	0.188*	
H19C	0.3113	0.2508	1.1478	0.188*	
C21	0.5326 (7)	0.1326 (4)	1.0908 (5)	0.092 (2)	
H21A	0.5768	0.1837	1.1089	0.139*	
H21B	0.5942	0.1086	1.0526	0.139*	
H21C	0.5225	0.0932	1.1313	0.139*	
C23	0.0528 (11)	−0.0661 (5)	0.8660 (4)	0.107 (3)	
H23A	−0.0202	−0.1095	0.8598	0.161*	
H23B	0.1467	−0.0864	0.8486	0.161*	
H23C	0.0247	−0.0173	0.8375	0.161*	
C24	−0.1127 (8)	0.0025 (6)	0.9863 (8)	0.153 (5)	
H24A	−0.1864	−0.0304	0.9611	0.229*	
H24B	−0.1193	0.0601	0.9702	0.229*	
H24C	−0.1285	−0.0005	1.0394	0.229*	

### Atomic displacement parameters (Å^2^)

**Table:**

	*U*^11^	*U*^22^	*U*^33^	*U*^12^	*U*^13^	*U*^23^
Ni1	0.0381 (3)	0.0439 (3)	0.0325 (3)	−0.0004 (2)	−0.0001 (3)	−0.0011 (2)
P1	0.0460 (8)	0.0455 (7)	0.0568 (8)	0.0043 (5)	−0.0126 (6)	−0.0108 (6)
Cl2	0.0715 (9)	0.0493 (6)	0.0394 (6)	0.0043 (6)	−0.0041 (6)	0.0024 (5)
P2	0.0516 (8)	0.0554 (8)	0.0588 (8)	−0.0134 (6)	−0.0078 (6)	0.0076 (6)
Cl1	0.0980 (12)	0.0663 (9)	0.0660 (9)	0.0286 (8)	−0.0186 (8)	0.0038 (7)
C1	0.040 (3)	0.052 (3)	0.032 (2)	−0.006 (2)	−0.0020 (18)	−0.0021 (19)
C11	0.048 (3)	0.067 (3)	0.034 (2)	0.011 (2)	0.005 (2)	−0.001 (2)
C7	0.038 (3)	0.049 (3)	0.038 (2)	0.006 (2)	0.0041 (19)	0.008 (2)
C6	0.047 (3)	0.037 (2)	0.037 (2)	−0.006 (2)	0.000 (2)	0.0006 (18)
C5	0.069 (4)	0.043 (3)	0.047 (3)	0.000 (2)	−0.010 (3)	0.000 (2)
C12	0.040 (3)	0.050 (3)	0.035 (2)	0.006 (2)	0.0009 (19)	0.001 (2)
C13	0.063 (3)	0.058 (3)	0.044 (3)	0.000 (3)	0.004 (2)	−0.003 (2)
C16	0.061 (4)	0.092 (5)	0.050 (3)	0.013 (3)	0.012 (3)	−0.013 (3)
C3	0.077 (4)	0.092 (4)	0.034 (3)	−0.016 (4)	0.007 (3)	0.008 (3)
C8	0.059 (3)	0.052 (3)	0.051 (3)	−0.003 (3)	0.009 (2)	0.009 (2)
C10	0.056 (3)	0.083 (4)	0.050 (3)	−0.006 (3)	0.014 (2)	0.013 (3)
C9	0.066 (4)	0.070 (4)	0.055 (3)	−0.009 (3)	0.006 (3)	0.016 (3)
C4	0.080 (4)	0.071 (4)	0.037 (3)	−0.002 (3)	−0.002 (3)	0.012 (2)
C2	0.050 (3)	0.076 (3)	0.039 (3)	−0.001 (3)	0.007 (2)	−0.004 (2)
C15	0.091 (5)	0.073 (4)	0.055 (3)	0.015 (4)	−0.005 (3)	−0.020 (3)
C14	0.094 (5)	0.052 (3)	0.051 (3)	−0.003 (3)	−0.006 (3)	−0.006 (2)
C17	0.038 (2)	0.045 (2)	0.042 (2)	0.0057 (19)	0.003 (2)	−0.006 (2)
N1	0.044 (2)	0.043 (2)	0.0351 (19)	0.0075 (17)	0.0027 (17)	−0.0007 (16)
C20	0.120 (6)	0.060 (4)	0.122 (6)	−0.024 (4)	−0.063 (6)	0.014 (4)
C22	0.155 (7)	0.066 (4)	0.080 (4)	−0.054 (4)	−0.041 (5)	0.024 (4)
C19	0.130 (7)	0.113 (6)	0.134 (7)	0.009 (6)	0.032 (7)	−0.069 (6)
C21	0.063 (4)	0.065 (4)	0.149 (7)	0.003 (3)	−0.045 (4)	−0.010 (4)
C23	0.151 (7)	0.094 (5)	0.078 (4)	−0.054 (5)	−0.051 (5)	0.005 (4)
C24	0.042 (4)	0.111 (7)	0.305 (17)	−0.021 (4)	0.022 (7)	−0.009 (9)

### Geometric parameters (Å, °)

**Table:**

Ni1—C1	1.891 (4)	C8—C9	1.405 (7)
Ni1—P2	2.1908 (15)	C8—H8	0.9300
Ni1—P1	2.1973 (14)	C10—C9	1.346 (8)
Ni1—Cl2	2.2443 (13)	C10—H10	0.9300
Ni1—N1	2.297 (4)	C9—H9	0.9300
P1—C21	1.796 (6)	C4—H4	0.9300
P1—C19	1.803 (7)	C2—H2	0.9300
P1—C20	1.818 (7)	C15—C14	1.391 (9)
P2—C24	1.790 (8)	C15—H15	0.9300
P2—C22	1.804 (6)	C14—H14	0.9300
P2—C23	1.819 (7)	C17—N1	1.279 (6)
Cl1—C5	1.752 (6)	C17—H17	0.9300
C1—C6	1.402 (7)	C20—H20A	0.9600
C1—C2	1.420 (7)	C20—H20B	0.9600
C11—C16	1.415 (8)	C20—H20C	0.9600
C11—C10	1.423 (8)	C22—H22A	0.9600
C11—C12	1.421 (6)	C22—H22B	0.9600
C7—C8	1.362 (7)	C22—H22C	0.9600
C7—C12	1.414 (6)	C19—H19A	0.9600
C7—N1	1.441 (6)	C19—H19B	0.9600
C6—C5	1.406 (7)	C19—H19C	0.9600
C6—C17	1.456 (6)	C21—H21A	0.9600
C5—C4	1.368 (8)	C21—H21B	0.9600
C12—C13	1.428 (7)	C21—H21C	0.9600
C13—C14	1.349 (7)	C23—H23A	0.9600
C13—H13	0.9300	C23—H23B	0.9600
C16—C15	1.339 (9)	C23—H23C	0.9600
C16—H16	0.9300	C24—H24A	0.9600
C3—C2	1.372 (8)	C24—H24B	0.9600
C3—C4	1.384 (8)	C24—H24C	0.9600
C3—H3	0.9300		
			
C1—Ni1—P2	89.63 (14)	C10—C9—H9	119.8
C1—Ni1—P1	86.35 (14)	C8—C9—H9	119.8
P2—Ni1—P1	159.73 (6)	C5—C4—C3	119.6 (5)
C1—Ni1—Cl2	177.94 (15)	C5—C4—H4	120.2
P2—Ni1—Cl2	89.94 (5)	C3—C4—H4	120.2
P1—Ni1—Cl2	93.37 (5)	C3—C2—C1	120.3 (5)
C1—Ni1—N1	80.57 (17)	C3—C2—H2	119.8
P2—Ni1—N1	99.30 (11)	C1—C2—H2	119.8
P1—Ni1—N1	99.62 (10)	C16—C15—C14	120.5 (5)
Cl2—Ni1—N1	101.48 (10)	C16—C15—H15	119.7
C21—P1—C19	101.4 (5)	C14—C15—H15	119.7
C21—P1—C20	102.4 (4)	C13—C14—C15	120.8 (6)
C19—P1—C20	101.6 (5)	C13—C14—H14	119.6
C21—P1—Ni1	116.6 (2)	C15—C14—H14	119.6
C19—P1—Ni1	116.8 (3)	N1—C17—C6	118.7 (4)
C20—P1—Ni1	115.7 (3)	N1—C17—H17	120.6
C24—P2—C22	102.5 (5)	C6—C17—H17	120.6
C24—P2—C23	103.7 (6)	C17—N1—C7	119.2 (4)
C22—P2—C23	100.5 (3)	C17—N1—Ni1	107.0 (3)
C24—P2—Ni1	111.3 (3)	C7—N1—Ni1	133.7 (3)
C22—P2—Ni1	120.1 (2)	P1—C20—H20A	109.5
C23—P2—Ni1	116.5 (3)	P1—C20—H20B	109.5
C6—C1—C2	118.6 (4)	H20A—C20—H20B	109.5
C6—C1—Ni1	116.0 (3)	P1—C20—H20C	109.5
C2—C1—Ni1	125.4 (4)	H20A—C20—H20C	109.5
C16—C11—C10	122.1 (5)	H20B—C20—H20C	109.5
C16—C11—C12	118.9 (5)	P2—C22—H22A	109.5
C10—C11—C12	119.0 (4)	P2—C22—H22B	109.5
C8—C7—C12	120.9 (4)	H22A—C22—H22B	109.5
C8—C7—N1	117.4 (4)	P2—C22—H22C	109.5
C12—C7—N1	121.7 (4)	H22A—C22—H22C	109.5
C1—C6—C5	119.1 (4)	H22B—C22—H22C	109.5
C1—C6—C17	117.7 (4)	P1—C19—H19A	109.5
C5—C6—C17	123.1 (5)	P1—C19—H19B	109.5
C4—C5—C6	121.4 (5)	H19A—C19—H19B	109.5
C4—C5—Cl1	118.6 (4)	P1—C19—H19C	109.5
C6—C5—Cl1	120.0 (4)	H19A—C19—H19C	109.5
C7—C12—C13	124.3 (4)	H19B—C19—H19C	109.5
C7—C12—C11	118.2 (4)	P1—C21—H21A	109.5
C13—C12—C11	117.4 (4)	P1—C21—H21B	109.5
C14—C13—C12	121.0 (5)	H21A—C21—H21B	109.5
C14—C13—H13	119.5	P1—C21—H21C	109.5
C12—C13—H13	119.5	H21A—C21—H21C	109.5
C15—C16—C11	121.2 (5)	H21B—C21—H21C	109.5
C15—C16—H16	119.4	P2—C23—H23A	109.5
C11—C16—H16	119.4	P2—C23—H23B	109.5
C2—C3—C4	120.9 (5)	H23A—C23—H23B	109.5
C2—C3—H3	119.5	P2—C23—H23C	109.5
C4—C3—H3	119.5	H23A—C23—H23C	109.5
C7—C8—C9	120.5 (5)	H23B—C23—H23C	109.5
C7—C8—H8	119.7	P2—C24—H24A	109.5
C9—C8—H8	119.7	P2—C24—H24B	109.5
C9—C10—C11	120.8 (5)	H24A—C24—H24B	109.5
C9—C10—H10	119.6	P2—C24—H24C	109.5
C11—C10—H10	119.6	H24A—C24—H24C	109.5
C10—C9—C8	120.5 (5)	H24B—C24—H24C	109.5
